# Cervical human papillomavirus infection and squamous intraepithelial lesions in rural Gambia, West Africa: viral sequence analysis and epidemiology

**DOI:** 10.1038/sj.bjc.6602736

**Published:** 2005-10-11

**Authors:** S R Wall, C F Scherf, L Morison, K W Hart, B West, G Ekpo, A N Fiander, S Man, C M Gelder, G Walraven, L K Borysiewicz

**Affiliations:** 1 Infection & Immunity, Henry Wellcome Research Institute, Wales College of Medicine, Cardiff University, Cardiff CF14 4XX, Wales, UK; 2 Obstetrics & Gynaecology, Wales College of Medicine, Cardiff University, Cardiff CF14 4XN, Wales, UK; 3 London School of Hygiene & Tropical Medicine, London WC1E 7HT, UK; 4 MRC Laboratories Farafenni, PO Box 273, Banjul, The Gambia; 5 Imperial College, London SW7 2AZ, UK

**Keywords:** HPV, SIL, DNA sequence, unselected, rural, Africa, vaccination

## Abstract

The development of effective strategies against cervical cancer in Africa requires accurate type specific data on human papillomavirus (HPV) prevalence, including determination of DNA sequences in order to maximise local vaccine efficacy. We have investigated cervical HPV infection and squamous intraepithelial lesions (SIL) in an unselected cohort of 1061 women in a rural Gambian community. Squamous intraepithelial lesions was diagnosed using cytology and histology, HPV was typed by PCR-ELISA of DNA extracts, which were also DNA sequenced. The prevalence of cervical HPV infection was 13% and SIL were observed in 7% of subjects. Human papillomavirus-16 was most prevalent and most strongly associated with SIL. Also common were HPV-18, -33, -58 and, notably, -35. Human papillomavirus DNA sequencing revealed HPV-16 samples to be exclusively African type 1 (Af1). Subjects of the Wolof ethnic group had a lower prevalence of HPV infection while subjects aged 25–44 years had a higher prevalence of cervical precancer than older or younger subjects. This first report of HPV prevalence in an unselected, unscreened rural population confirms high rates of SIL and HPV infection in West Africa. This study has implications for the vaccination of Gambian and other African populations in the prevention of cervical cancer.

It is well established, on a cellular and epidemiological basis, that the sexual transmission of high oncogenic risk (HR) human papillomavirus (HPV) types is the essential prerequisite for cervical carcinogenesis (zur Hausen, 1977; IARC_Monographs, 1995; Muñoz and Bosch, 1996; Walboomers *et al*, 1999). Cervical cancer is the most common malignancy in West African women (Koulibaly *et al*, 1997; Bah *et al*, 2001) but accurate, population-based HPV prevalence data are lacking. Many therapeutic and prophylactic vaccines against HPV infection are in development, with some now entering phase III trials (Bosch *et al*, 2001; Nardelli-Haefliger *et al*, 2003; Taira, 2004; Tomson *et al*, 2004; Christensen, 2005; Gravitt and Shah, 2005; Maclean *et al*, 2005). Such vaccines are essential in developing countries where 80% of new cervical cancer cases occur but where screening and treatment of precancerous lesions is not available (Kitchener and Symonds, 1999; Lazcano-Ponce *et al*, 1999). Risk factors in the spread of genital HPV infection vary between populations, and include genetic variation in human leukocyte antigen (HLA) types resulting in differential susceptibility to HPV infection and socio-demographic factors such as sexual behaviour or age (Burk *et al*, 1996; Koutsky, 1997; Svare *et al*, 1998; Herrero, 1999). Consequently, prevalence of cervical cancer in industrialised nations cannot be assumed to apply in the developing world; local characterisation of HPV patterns is essential. Human papillomavirus-16 is the most prevalent type in cervical cancers worldwide (Bosch *et al*, 1995b), although regional variation in HR HPV types does occur (Bosch *et al*, 1995a; Herrero *et al*, 2000; Castellsague *et al*, 2001; Mayaud *et al*, 2001; Xi *et al*, 2003). HPV DNA sequence must therefore be defined to ensure vaccine efficacy and prevent the selective emergence of rare, virulent variants. Here we describe such detailed HPV analysis in a rural Gambian community, presenting the largest point-prevalence study to date in a rural unselected sub-Saharan African population.

## METHODS

### Population

Samples were collected during a large reproductive morbidity survey in rural Gambia between January and July 1999 as described elsewhere (Walraven *et al*, 2001). Briefly, the MRC has run a continuous demographic surveillance system in 40 villages surrounding the provincial town of Farafenni since 1981. All women aged 15–54 years in 20 of these villages were approached and invited to participate. The study was approved by the ethics committee of The Gambian Government/MRC Laboratories (SCC proposal 755). All work was conducted in accordance with the Helsinki Declaration of 1975 as revised in 1983. After obtaining informed consent. A total of 1348 women (72% of those eligible) were seen and examined in dedicated village clinics.

### Sampling

**Figure 1:**
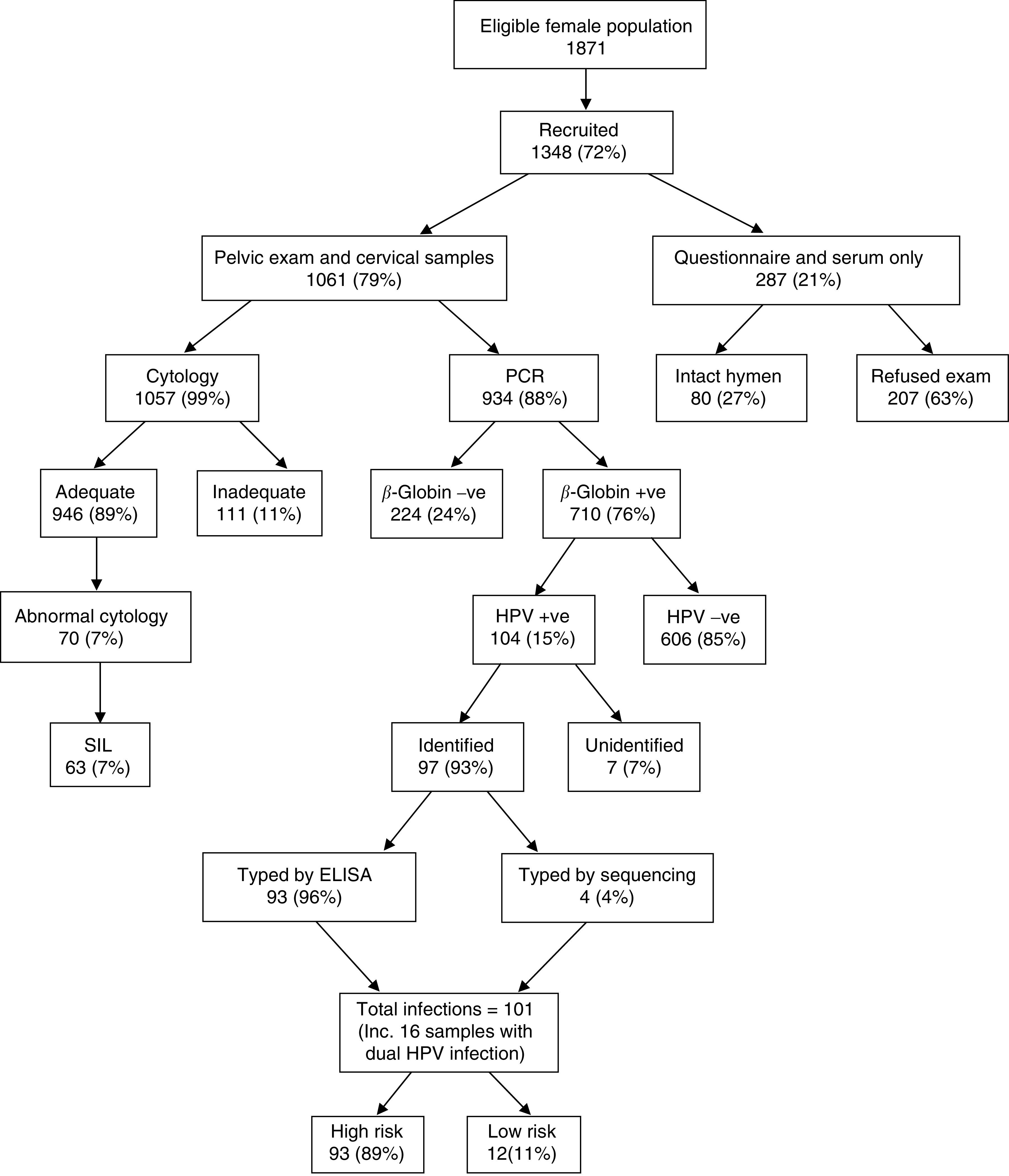
Overview of gambia reproductive morbidity study.

As part of the full gynaecological assessment, all consenting subjects who did not have an intact hymen underwent a vaginal speculum examination (Figure 1). Cervical smears were taken with an Aylesbury spatula for cytology (Wolfendale *et al*, 1987) fixed, stored and transported to Cardiff. Human papillomavirus sampling was performed by inserting the brush-sampler into the cervical canal and rotating it through 360°. This brush was placed in transport medium immediately (Digene Corporation, Gaithersburg, MD, USA), stored at −70°C and frozen samples transported to Cardiff for analysis.

### Cytology

Women with abnormalities were offered further assessment and treatment with Large Loop Excision of the Transformation Zone (LLETZ) or knife cone biopsy. Histology and cytology results were subjected to clinical review to decide on a final diagnosis viz low- or high-grade squamous intraepithelial lesions (LSIL or HSIL) (NCI_Workshop, 1989), microinvasive or invasive carcinoma of the cervix.

### Human papillomavirus detection and typing

DNA was extracted from cervical brush samples by freeze–thaw from −70°C, boiling for 10 min followed by rapid cooling on ice. Human papillomavirus DNA was detected by PCR using the consensus HPV primers GP5+ and GP6+ (biotinylated) (Jacobs *et al*, 1997). In tandem, for each sample a PCR was also set up to amplify the human *β*-globin gene in order to verify the presence of an adequate DNA sample and the absence of PCR inhibitors. The presence of HPV and *β*-globin DNA was visualised on an agarose gel. Human papillomavirus typing was preformed by enzyme linked immuno-sorbent assay (ELISA) using streptavidin coated microtitre plates to capture PCR product (Jacobs *et al*, 1997). Initially, this involved two cocktails of digoxigenin labelled probes, the first including those types commonly associated with cervical cancer (HPV-16, -18, -31, -33, -35, -39, -45, -51, -52, -56, -58, -59, -66 and -68). The second cocktail included those types associated with benign genital lesions (HPV-6, -11, -40, -42, -43 and -44). Detection was performed using an alkaline phosphatase conjugated antidigoxigenin fab fragment. If positive for either of the two probes, a further ELISA was performed using the full spectrum of relevant (HR) or low risk (LR) individual probes.

### Sequencing

Samples that could not be identified by ELISA were characterised by DNA sequencing. Selected HPV positive samples were also sequenced to confirm typing accuracy and to detect any relevant sequence variations in known HPV types. Sequencing was carried out on a Beckman Coulter CEQ2000 Automated Sequencer, using protocols and reagents supplied by the manufacturer. Human papillomavirus DNA sequencing utilised the MY09/MY11 consensus primer pair as the product of these (≈455 bp) is a more suitable target for sequencing than that of GP5+/6+ (≈140 bp). Furthermore, MY09/11 primers amplify a region containing highly conserved amino acids, which simplifies sequence alignment in HPV classification (Chan *et al*, 1995). An NCBI BLAST (http://www.ncbi.nlm.nih.gov/BL AST/) search was carried out for each of the consensus strands produced by sequencing at least twice in each direction. Alignment analysis was carried out using the Lasergene suit of software (DNA^*^ Inc., Madison, WI, USA). The sequences most homologous to the Gambia samples are listed in Table 2 with the percentage DNA similarity and number of differences in both amino acids (coding) and DNA only (noncoding) in the MY9/11 amplified region between the Gambia samples and the closest BLAST hits.

### Statistical analysis

All data were double-entered, checked and cleaned using Epi-Info 6.4 with subsequent analysis performed in STATA 7 (Statacorp. Texas). Frequency distributions of HPV infection and different HPV types were calculated. Socio-demographic characteristics and other morbidities were examined as risk factors for HPV infection by cross tabulation and *χ*^2^ tests. To adjust for any confounding effects, each potential risk factor was included in a logistic regression model with age group, marital status and ethnic group. The same procedure was followed for SIL. There were significant differences in the distribution of ethnic groups between the sample and the eligible population and differing disease prevalence between ethnic groups (Table 1). An adjusted prevalence was therefore calculated using the prevalence within each ethnic group and the distribution of ethnic groups in the eligible population.

## RESULTS

Of 1871 eligible women, 1348 (72%) participated in the study (Figure 1) and 1061 women consented to full gynaecological examination. Of participants aged 15–24 years, 30.7% (147 out of 478) were unmarried, 80 of whom consented to intimate examination and had an intact hymen (Figure 1). These women were not examined internally by speculum and no cervical specimen was collected. No HPV sample was taken from a further 64 women examined by speculum and 63 samples were lost in transit. The *β*-globin human housekeeping gene was amplified in 710 out of 934 (76%) samples; consequently, only these samples were suitable for HPV analysis. The *β*-globin negative samples may have been incorrectly stored or damaged during transfer to the UK, prevalence figures are therefore based upon the 710 *β*-globin positive samples. Of the three main ethnic groups, Wolof women were slightly under-represented (38.7% of eligible population *vs* 32.1% of those with adequate cytology and 34.1% of those with adequate HPV samples) and Mandinka women were slightly over-represented (43.9% of eligible population *vs* 49.5% of those with adequate cytology and 46.2% of those with adequate HPV samples). Younger women aged 15–24 years were under-represented (27.9 and 25.9% in those with adequate cytology and adequate HPV samples, respectively, compared with 39.1% in the eligible population).

### Human papillomavirus prevalence

**Figure 2:**
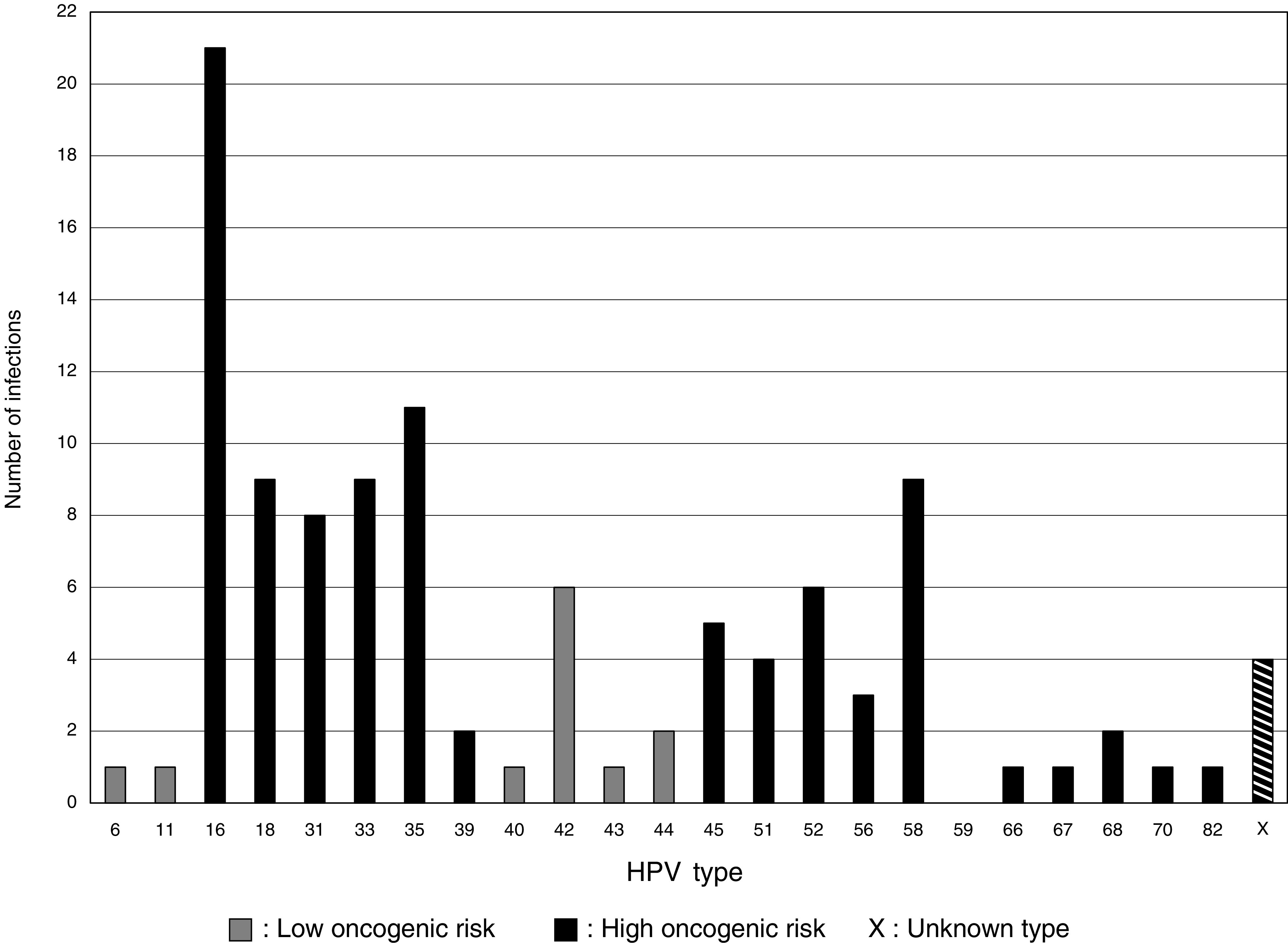
Total of all HPV types (inc. multiple infections).

Human papillomavirus infection was present in 95 of 710 adequate samples (crude prevalence 13.4% (95% CI: 10.96–16.11%)). Adjusting HPV prevalence for under-representation of Wolof subjects reduced prevalence slightly to 13.0%. Human papillomavirus typing by PCR-ELISA was successful in 84 samples revealing 6 different LR and 13 different HR types (Figure 2). Dual cervical HPV infection was found in 16 subjects. HPV-16 (21 out of 109 (19%)) and HPV-35 (11 out of 109 (10%) were most common. Other prevalent types were HPV-18, -33 and -58 (each 9/109 (8%)), HPV-31 (8/109 (7%)) and HPV-42 (6/109 (6%)).

### DNA sequencing

Human papillomavirus DNA was amplified with the GP5+/6+ primers in 11 samples but were negative by ELISA, and therefore contained HPV types not included in the probe cocktails. These samples were sequenced with the MY 09/11 primers; five had either degenerated in storage or would not amplify, two contained multiple templates and were impossible to sequence, while four were successfully sequenced and identified but none were infected with novel types, merely types not included in the ELISA probe cocktail (Table 2).

DNA sequencing was conducted on samples successfully identified by ELISA as HPV types associated with HSIL. All HPV-16 samples sequenced showed DNA homology in the amplified region with a variant of HPV-16 African type 1 (Af1, Accession No. AF536180) (Table 2). Both HPV-18 samples sequenced displayed protein sequence homology to HPV-18 variants from Benin, West Africa (Accession Nos. U45894 and U45892) (Table 2). HPV-31 from our study showed a number of polymorphisms and silent mutations compared to the reference sequence (Accession No. J04353), but insufficient to be a novel type (Bernard, 2005) (Table 2). All HPV-33 samples sequenced displayed protein sequence homology to the reference HPV-33 strain (Accession No. M12732) (Table 2). The sequence of HPV-58 from our study was identical to a variant isolated in the West African nation of Mali (Table 2).

### Cytology and histology

The overall crude prevalence of cervical precancer was 6.7% (63/946 adequate smears (95% CI: 5.2–8.4%)), or 6.5% after adjusting for the under-representation of Wolof women Cytological abnormalities were confirmed by histology in 55.6% (35/63) of cases with HSIL present in 2.3% (*n*=22; 16 confirmed by histology), LSIL were found in 3.3% (*n*=31; 19 confirmed by histology) and atypical squamous cells of uncertain significance (ASCUS) in 1.1% (*n*=10). A single case of invasive cervical cancer was diagnosed by clinical and cytological criteria but the woman sadly died before the histology could be confirmed; it was included in the HSIL analysis.

### Epidemiology

Risk factors for cervical precancer included age (*χ*^2^ *P*=0.03) and cervical HPV infection. Rates of SIL were highest at ages 25–34 years (22 out of 265 (8%)) and 35–44 years (24/268 (9%)), intermediate at ages 45–54 (9/150 (6%)) and lowest at ages 15–24 (8/263 (3%)). In subjects where both adequate cytology/histology and HPV analysis were available, 574/612 (93%) had normal cytology, 38 out of 612 (6%) had SIL and seven out of 612 (1%) had ASCUS. Of the subjects with normal cytology 525/574 (91%) were negative for HPV while in those with SIL/ASCUS 20/38 (53%) had HR HPV types. Of subjects with HSIL, 13/15 (87%) had HR HPV types, although the other two were negative for HPV. Among subjects with HSIL, HPV-16, HPV-33 and HPV-58 were most common, each found in 3/15 (20%). HPV-16 was found in the single invasive cancer case. HPV-18, -31, -35, -39 and -45 occurred once each among subjects with HSIL. Of 30 subjects with LSIL/ASCUS, seven were infected with HR HPV types; of subjects with normal cytology 66/576 (12%) had HPV infection.

No associations were found between HPV and other current sexually transmitted infections (STI) (Table 1). Moderate or severe anaemia was associated with HPV infection, but not significantly. Similarly, abnormal vaginal bleeding or discharge, pelvic masses and body mass index showed no significant associations with HPV infection. Of 1110 subjects, only 61 (6%) used modern methods of contraception, too few to examine associations with HPV. Among socio-demographic factors, only the subject's ethnic origin was significantly associated with HPV infection, with Mandinka (14%) and Fula (21%) women having a higher risk of cervical HPV infection than Wolof subjects (8%) (Table 1).

## DISCUSSION

This first unselected study of cervical HPV and SIL in an unscreened, rural West-African population of its kind shows HPV infection to be common at 13.4% (adjusted=13.0%) with the majority HR types. Although younger subjects were under-represented, such an age profile is comparable with other studies. The high cervical HPV infection prevalence and SIL are in agreement with Gambian Cancer Registry data (Koulibaly *et al*, 1997; Bah *et al*, 2001). Comparable prevalences of 14% have been observed in two cytologically normal populations in Senegal, the only nation on which The Gambia borders (Astori *et al*, 1999; Xi *et al*, 2003). A recent study from Nigeria, West Africa, of an unselected population of similar size typed by GP5+/6+ PCR-ELISA, found an HPV prevalence of 26% (Thomas *et al*, 2004). However, the population in Thomas *et al* was urban (Ibadan, pop. >1M) and the higher HPV prevalence may be explained by differing sexual behaviour as well as the much higher prevalence of LR HPV types in the Nigerian study than in our study.

Much higher HPV prevalence figures have been reported in recent unselected studies from Eastern and Southern Africa, ranging from 34% in rural Zimbabwe (Baay *et al*, 2004) to 44% in urban Kenya (De Vuyst *et al*, 2003). Human immunodeficiency virus (HIV) infection and concomitant immune suppression is an acknowledged cofactor in the progression of cervical cancer (Feingold *et al*, 1990; Moscicki *et al*, 2004a, 2004b) and such high HPV prevalence may be due to the high rates of HIV infection in these regions (UNAIDS, 2004). The Gambia has one of the lowest HIV infection rates in Africa (Ramsay, 1993, [73]Da Costa, 1994; Schim van der Loeff *et al*, 2003). Multiple cervical HPV infection is common in Africa (Chabaud *et al*, 1996; Castellsague *et al*, 2001; Gravitt *et al*, 2002; Stanczuk *et al*, 2003; Xi *et al*, 2003; Baay *et al*, 2004) yet we encountered no more than two co-infections, perhaps because of the low HIV rates in The Gambia (UNAIDS, 2004). Multiple HPV infection have been linked to HIV immunosuppression (Levi *et al*, 2002a, 2002b; Moscicki *et al*, 2004a) and in Zimbabwean HIV infected individuals may exhibit quintuple HPV infection (Baay *et al*, 2004).

High oncogenic risk HPV can be detected in >99.7% of high-grade pre-invasive and invasive cervical lesions (Walboomers *et al*, 1999). In this study, HPV was found in 87% of subjects with high-grade lesions, indicating a high degree of accuracy for HPV detection. Failure to detect HPV in two subjects with HSIL may be due to integration of viral DNA into their genomic DNA and the consequent deletion of the target site for the consensus PCR primers. HPV shows a wide type distribution (Figure 2) (eight types associated with HSIL), but an even distribution among subjects. HPV-16, the most common HR type worldwide (Bosch, 1995a, 1995b; Muñoz *et al*, 2003) is the most common type in this study, showing the strongest association with precancer.

The HR type HPV-35 was the second most prevalent type (10%) in this study and meta-analysis of worldwide HPV prevalence studies shows low overall prevalence of HPV-35 (≈2%), even in Africa (Clifford *et al*, 2003b). Three selective urban Senegalese studies found a very low prevalence of HPV-35 ranging from no HPV-35 positive samples (Astori *et al*, 1999; Chabaud *et al*, 1996) to 1% in the most recent (Xi *et al*, 2003). Inconsistency between studies examining Senegal and Gambian populations with overlapping ethnic groups, religious practices and trade routes initially appears incongruous. However, the GP5+/6+ primers used in the Gambian study are estimated to be 5000 times more sensitive in the detection of HPV-35 (Qu *et al*, 1997) than the MY09/MY11 primer pair used in the Xi and Astori studies. Therefore, the true HPV-35 prevalence in these Senegalese studies is likely to have been underestimated. This is supported by other African studies where HPV-35 is among the four of the most prevalent types in studies not exclusively using the MY09/11 primer pair for HPV typing (Castellsague *et al*, 2001; De Vuyst *et al*, 2003; Baay *et al*, 2004; Naucler *et al*, 2004; Thomas *et al*, 2004). The Nigerian population displays a similar distribution of HR types to the Gambian except that HPV-16 and -35 are jointly the most prevalent types in Nigerian study (Thomas *et al*, 2004). It thus appears that HPV-35 is prevalent throughout sub-Saharan Africa but the extent of this prevalence is underestimated by the use of the MY09/11 primer pair. This indicates the importance of methodology in the design of viral epidemiological studies. Of the 13% (12/93) of samples positive for LR HPV, HPV-42 was most prevalent, accounting for 50% of LR types and 6% of all HPV types. This is in contrast to the USA and Europe where HPV-6 and HPV-11 are the dominant LR types (Muñoz *et al*, 2003) and most other African studies, where HPV-53 and -54 are the dominant LR types (Castellsague *et al*, 2001; De Vuyst *et al*, 2003; Xi *et al*, 2003). HPV-42 was the most prevalent LR type and overall the most common HPV type in Nigeria (Thomas *et al*, 2004), increasing the overall HPV prevalence, with a LR : HR HPV ratio of 35 : 65 compared to 11 : 89 in our Gambian study.

Human papillomavirus is classified into types, subtypes and variants by comparative DNA homology based upon the L1 outer capsid. To define a new HPV type it must have less than 90% L1 DNA homology to any previously defined types. Human papillomavirus types can be further characterised into subtypes, 90–98% L1 DNA homology, and variants (either >98% L1 DNA homology or oncogene variations). No HPV samples in this study were found to be novel types after identification by sequencing, although some were unusual subtypes or variants (Table 2). The DNA sequences of HPV-16, -18, -33 and -68 revealed HPV variants found by other African studies. In particular, the Gambian HPV-68 samples are an exact DNA match to an HPV-68 subtype first isolated from a study in neighbouring Senegal (Astori *et al*, 1999). Our study also confirms the importance of HPV-16 Af1, the most prevalent of the HPV-16 subtypes in Africa (Yamada *et al*, 1997). Gambian HPV-18 and HPV-33 variants are homologous to those found in Benin, Uganda and Guinea (Table 2). Thus, with the non-HPV-16 samples associated with HSIL in this study, a number of polymorphisms are observed compared to the reference types but most are identical or similar to types found in other African studies (Table 2).

Histologically confirmed cervical neoplasia prevalence in our study was 6.7% (adjusted=6.5%). Diagnostic accuracy was improved by classifying subjects with abnormal cytology but normal histology as ‘normal cervix’. The prevalence of abnormal cytology alone was slightly higher at 7.4% (70/946 adequate smears). Other investigators have used a cytological screening to identify cervical disease providing a less accurate prevalence of SIL (Herrero *et al*, 2000; Castellsague *et al*, 2001).

In this study, HPV infection was not associated with age, parity or concurrent STI. The even distribution of HPV prevalence at different ages is unusual though observed in other developing countries such as India (Franceschi, 2005) and Argentina (Matos *et al*, 2003). Studies from industrialised countries show a high HPV prevalence among women under 25 years declining with age (Sellors *et al*, 2000; Giuliano *et al*, 2001). This may be explained by study bias since data are commonly collected from such highly selective populations as college students or clinic attendees. Our study, however, concerns a nonselective population with minimal migration; differing sexual behaviour in different cultural environments may contribute to the variable age-profiles. Certain ethnicities were associated with higher risk of cervical HPV infection in Gambia, a decreased risk being seen in Wolof women compared with Mandinka and Fula subjects, perhaps due to differing patterns of sexual behaviour. Another possibility is genetic variation in susceptibility to HPV infection, for example, due to differences in HLA types while differences in female genital cutting (FGC) between the ethnic groups suggest a further mechanism. The Wolof are the only ethnic group in our study that do not practice FGC and may thereby be less susceptible to HPV infection. A previous study of this group found lower prevalences of HSV-2 (herpes simplex virus) and bacterial vaginosis among nongenitally cut women (Morison *et al*, 2001).

The ideal treatment strategy for this rural Gambian population would involve the use of a prophylactic vaccine to prevent cellular viral entry. Current prophylactic vaccine formulations require three needle injections, cold storage and offer only type specific protection (Koutsky *et al*, 2002; Harper *et al*, 2004). In rural Gambia with a spread of prevalent HR HPV types, where funding is short supply, this is impractical. However, vaccines currently in development include oral based prophylactic vaccines (Baud *et al*, 2004; Berg *et al*, 2005; Sasagawa *et al*, 2005), which will be easier to store and administer and thus economically viable.

The ethnic groups in our study population have not previously been studied using these methods. Other studies in African populations using the same methods to those used in our study (La Ruche *et al*, 1998; Bayo *et al*, 2002; Baay *et al*, 2004; Naucler *et al*, 2004; Thomas *et al*, 2004) show differing LR/HR ratios and disease associations. This suggests that in addition to HPV infection host factors are central in the development of cervical cancer. Our findings strengthen existing data from developing nations, showing widespread cervical HPV infection of a broader spectrum than found in industrialised nations with HPV-16 most common. An effective vaccine for the Gambia must be multivalent and include the other HR types prevalent in this representative rural population including HPV-18, -58 and -33. HPV-35 was not found in high-grade lesions and evidence for its oncogenicity is limited. However, as the second most prevalent type in this study with close homology to HPV-16, it may be worth including in any potential vaccine.

## Figures and Tables

**Table 1:** Association between cervical HPV infection and socio-demographic, behavioural and infectious factors in the Gambia Reproductive Morbidity Study

	**HPV infected/total (%)**	**Crude OR (95%CI)**	***χ*^2^ *P*-value**	**Adjusted OR^a^ (95% CI)**	***P*-value^b^**
*Age group*					
15–24	27/184 (15%)	1		1	
25–34	22/213 (10%)	0.67 (0.37–1.22)		0.69 (0.37–1.29)	
35–44	27/191 (14%)	0.96 (0.54–1.7)		1.10 (0.60–2.04)	
45–54	19/122 (16%)	1.07 (0.57–2.02)	0.461	1.13 (0.57–2.25)	0.387

*Ethnic group* c					
Wolof	20/242 (8%)	1		1	
Mandinka	47/328 (14%)	1.86 (1.07–3.22)	0.002	1.76 (1.00–3.09)	
Fula	27/129 (21%)	2.94 (1.57–5.48)		2.85 (1.51–5.40)	0.005

*Marital status* c					
Monogamous	40/266 (15%)	1		1	
Polygamous	51/416 (12%)	0.79 (0.50–1.23)		0.84 (0.51–1.36)	
Divorced/widowed	3/22 (14%)	0.89 (0.25–3.15)	0.582	0.84 (0.23–3.07)	0.772

*Parity*					
Nulliparous	9/56 (16%)	1		1	
Para 1–3	29/205 (14%)	0.86 (0.38–1.94)		0.85 (0.37–1.96)	
Para 4–7	40/322 (12%)	0.74 (0.34–1.63)		0.83 (0.33–2.08)	
Para 8+	17/127 (13%)	0.81 (0.34–1.94)	0.871	0.65 (0.23–1.82)	0.844

*Sexually active in last 3 months*					
Yes	62/497 (12%)	1		1	
No	21/133 (16%)	1.31 (0.77–2.25)		1.23 (0.70–2.19)	
No answer	12/80 (15%)	1.23 (0.63–2.42)	0.549	1.19 (0.58–2.41)	0.731

*Genital prolapse*					
Absent	59/377 (16%)	1		1	
Present	36/329 (11%)	0.66 (0.42–1.03)	0.067	0.73 (0.46–1.16)	0.183

*HSV2 serology*					
Negative	53/444 (12%)	1		1	
Positive	40/244 (16%)	1.45 (0.93–2.26)	0.102	1.27 (0.78–2.08)	0.343

*Current STI* d					
Absent	81/605 (13%)	1		1	
Present	10/73 (14%)	1.03 (0.51–2.08)	0.941	0.77 (0.36–1.65)	0.494

*Endogenous infection* e					
Absent	48/328 (15%)	1		1	
Present	45/319 (14%)	0.96 (0.62–1.49)	0.848	1.00 (0.63–1.59)	0.985

*Anaemia* f					
Absent	41/322 (13%)	1		1	
Mild	33/272 (12%)	0.95 (0.58–1.54)		0.86 (0.52–1.43)	
Moderate/severe	18/96 (19%)	1.58 (0.86–2.91)	0.237	1.45 (0.77–2.73)	0.319

a Adjusted for age group, marital status and ethnic group.

b From likelihood ratio test adjusting for age group, marital status and ethnic group.

c Five single women (one had HPV) and 11 women of other ethnic groups (one had HPV) were excluded from this analysis because of small numbers.

d Chlamydia trachomatis, Trichomonas vaginalis, positive Syphilis serology.

e Candida culture positive, Bacterial vaginosis (Nugent's criteria).

f Anaemia: mild=Hb<11 (pregnant) Hb<12 (nonpregnant), moderate/severe=Hb<9 (pregnant), Hb<10 (nonpregnant).

**Table 2:** Genetic comparison of HPV DNA sequences in the Gambia Reproductive Morbidity Study compared to closest Genbank entries

				**Homology to Gambian sample(s)**		
**RMS Gambia sample**	**Genbank accession number**	**Isolate number (type)**	**Origin**	**% DNA similarity**	**Noncoding variations**	**Coding variations**
*Samples identified by sequencing*						
2006D	U01532	AE2 (MY9/11)	USA	100	0	0
	U12481	IS039 (MY9/11)	Argentina	99.8	1	0
	AF293961	AE2/IS039 (Full)	New York, USA	99.5	2	0
	AB027021	HPV-82 (Ref.)	Japan	89.0	38	8
2712F	D21208	HPV-67 (Ref.)	NK	98.3	4	3
	U12492	(MY09/11)	NK	97.8	6	3
2781F	U01535	AE1 (MY09/11)	USA	99.5	2	0
	U21941	HPV-70 (Ref.)	Sweden	99.3	3	0
	U12476	CP141 (MY09/11)	New Mexico, USA	99.1	3	1
	U12486	LVX160 (MY09/11)	Indigenous Amazonian	99.1	3	1
2919F	AF538717	SDL105 (MY09/11)	Minesota, USA	99.0	4	0
	Y17206	GA115 (MY09/11)	Senegal^c^	99.8	1	0
	M73258	ME180 (Cell Line)	NK	98.3	5	2
	U45934	IS362 (MY09/11)	Germany	97.8	7	2
	X67161	HPV-68 (Ref.)	NK	93.0	22	7

*Samples identified by PCR-ELISA*						
HPV-16	AF536180	Af1 variant (Full)	Africa	100	0	0
	U34188	OR7587 (L1)	USA	100	0	0
	AF472508	Af1 Variant (Full)	Africa	99.8	1	0
	U34189	OR7632 (L1)	USA	99.8	1	0
	U34183	OR6106 (L1)	USA	99.5	1	1
	AF472509	Af2 (Full)	Africa	99.3	2	1
	AF134178	GU2 (L1)	NK	99.3	2	1
	U37217	(L1+L2)	Zaire^d^	99.3	2	1
	U34186	OR7145 (L1)	USA	99.3	2	1
		HPV16R (Ref.)	Composite	99.0	4	0
HPV-18	U45894	IS172 (MY09/11)	Benin^c^	100	0	0
	U45892	IS168 (MY09/11)	Benin^c^	99.8	1	0
	U45893	IS768 (MY09/11)	Uganda^d^	99.3	2	1
		0069A^a^ (MY09/11)	Gambia	99.5	2	0
	X05015	HPV-18R (Ref.)	Brazil	97.6	8	2
HPV-31	U37410	(L1 & L2)	NK	99.0	3	1
	J04353	HPV-31 (Ref.)	NK	98.6	5	1
HPV-33	U45897	IS827 (MY09/11)	Guinea^c^	99.8	1	0
		0184C^b^ (MY09/11)	Gambia	99.8	0	1
	M12732	HPV-33 (Ref.)	NK	99.8	1	0
HPV-58	U45928	IS417 (MY09/11)	Mali	100	0	0
	AY101598	Bsb-2 (MY09/11)	Brazil	99.7	0	1
	U45929	IS404 (MY09/11)	Mali	99.2	1	2
	D90400	HPV-58 (Ref.)	Japan	98.9	1	3

a Sample from this study differing in two noncoding bases from the other HPV-18 samples sequenced.

b Sample from this study differing in one coding base from the other HPV-33 samples sequenced.

c West Africa.

d Central Africa.
